# Bulbar “Red Flag” Symptoms and a 21‐Year Diagnostic Delay in Adult Arnold–Chiari Type II Malformation With Tracheoesophageal Fistula and Syringomyelia: A Case Report

**DOI:** 10.1002/ccr3.73023

**Published:** 2026-07-03

**Authors:** Zahabia Adnan, Yashfa Rajput, Chanchan, Marium Fawad, Muhib Ullah Azhar, Abedin Samadi, Ahmed Asad Raza

**Affiliations:** ^1^ Jinnah Sindh Medical University Karachi Pakistan; ^2^ Kabul University of Medical Sciences, Abu Ali Sina Kabul Afghanistan

**Keywords:** Arnold–Chiari Malformation, cerebellum, posterior fossa decompression, syringomyelia, tracheoesophageal fistula

## Abstract

The simultaneous occurrence of Arnold–Chiari Malformation Type II (ACM II), syringomyelia, and an acquired tracheoesophageal fistula (TEF) in a young adult is exceptionally rare and represents a major diagnostic challenge. Arnold–Chiari Malformation II is characterized by the herniation of cerebellar structures through the foramen magnum. It usually presents in infancy in association with myelomeningocele; co‐existing TEF and syringomyelia in an adult, as in our patient, is seldom documented. Notably, the present case represents a Chiari II phenotype without spinal dysraphism, lying outside the classic definition that requires an associated myelomeningocele. A 21‐year‐old male presented with a two‐year history of progressive neurological and oropharyngeal symptoms. He also reported nasal regurgitation, cervicogenic vertigo, photophobia, and hoarseness. Neurological examination revealed multidirectional nystagmus, optic atrophy in the right eye, right cerebellar signs, fasciculations, and fundoscopy showed a filled disc and temporal margin blurring in the right eye. MRI of the brain and cervical spine revealed a 10.3 mm herniation of the cerebellar tonsils below the foramen magnum, typical of ACM Type II with syringomyelia. The brain CT scan identified a tracheoesophageal fistula above the carina with a 0.5 cm communication and a diverticulum on the right side of the trachea. Initially, the patient was treated symptomatically with antihistamines, antibiotics, and nasal sprays. The patient was also given multidisciplinary consultation for neurosurgical decompression and underwent posterior fossa decompression surgery, including suboccipital craniectomy and C1 laminectomy, to relieve compression at the foramen magnum and restore cerebrospinal fluid flow. This case underscores an unusual constellation of ACM Type II with extracranial complications and highlights the diagnostic importance of neuroimaging in complex craniospinal presentations.

## Key Clinical Message

Adult presentation of Arnold–Chiari Malformation Type II with simultaneous syringomyelia and acquired tracheoesophageal fistula is exceptionally rare. Timely neuroimaging, multidisciplinary collaboration, and early surgical intervention are crucial to prevent progressive neurological decline, aspiration‐related complications, and long‐term disability in such complex craniospinal cases.

## 1 Introduction

Arnold–Chiari malformations (ACM) constitute a group of congenital malformations concerning the hindbrain caused by distal descent of the tonsil and by other brainstem structures into the cervical spinal canal through the foramen magnum [1]. These disorders arise due to defective posterior fossa development and abnormal cerebrospinal fluid (CSF) dynamics during embryogenesis [2]. The four classical subtypes, Classes I to IV, are defined according to the degree of herniation and associated neural tube defect [3]. Among these types, Type II ACM, or classic Chiari malformation, is the most common variant found among the pediatric population and is usually associated with myelomeningocele, hydrocephalus, and other neural tube anomalies [4]. There's an exceedingly rare occurrence of diagnosis made in adults, which makes treatment difficult [5].

In ACM type II, the downward displacement of the vermis, medulla, and fourth ventricle distorts the brainstem and the upper cervical cord to obstruct CSF circulation, resulting in hydrocephalus, syringomyelia, and cranial‐nerve deficits [6]. The pathophysiology of this disease comes from various factors including small posterior volume, abnormal pressure gradient of CSF, and tractional forces along the neuroaxis [7]. Most cases are recognized in infancy or early childhood because of obvious neurological or developmental anomalies. However, late presentation or adult cases such as in the present one are rare and may present with subtle or progressively increasing signs of headache, limb weakness, ataxia, dysphagia, or dysarthria, often leading to misdiagnosis or delayed appreciation [8].

Syringomyelia is formed in the majority of ACM cases. It is a chronic disorder due to cystic formation in the spinal cord by the disturbed flow of CSF at the craniocervical junction [9]. Clinically, syringomyelia is evidenced as dissociated sensory loss, muscle wasting, spasticity, and gait disturbance. The occurrence of syringomyelia in ACM Type II dramatically changes prognosis and treatment planning. Continuous compression at the foramen magnum can encourage further syrinx formation, causing a build‐up of neurological deficits.

It is rare and quite odd in the present case to find an acquired tracheoesophageal fistula with ACM Type II and accompanying syringomyelia. A tracheoesophageal fistula is the abnormal communication between the trachea and esophagus, which can be congenital or acquired due to infection, trauma, malignancy, or prolonged intubation [10]. A few studies report the possible association between craniospinal malformations and foregut anomalies such as TEF. The embryological basis for this is a shared disruption of mesodermal and endodermal development affecting both neural tube and foregut structures. Alternatively, the existence of such comorbidities could be coincidental but hold immense clinical importance due to the increased morbidity sustained from recurrent aspiration and malnutrition as a consequence of respiratory compromise [11].

In adults who present with neurological symptoms or have had a history of such symptoms, diagnostic imaging, especially MRI, plays a pivotal role in revealing hindbrain malformations and associated problems [12]. Quick diagnosis is important as posterior fossa decompression halts the progression of the disease and offers beneficial neurological outcomes. Such cases require multidisciplinary management for optimal results since associated anomalies such as TEF present further complexity for the management involving neurosurgery, thoracic surgery, and rehabilitation.

## 2 Case History

A 21‐year‐old male, a known case of pulmonary tuberculosis with no other comorbidities, presented with a 2‐year history of progressive right‐sided weakness and a 1‐year history of difficulty in swallowing and speech. The illness began insidiously 2 years earlier with weakness and numbness in the right upper and lower limbs. Over time, the weakness gradually worsened, leading to difficulty in performing daily activities. The patient also reported imbalance, frequent slipping while walking, chronic right‐sided headaches, dizziness, vertigo on turning his head, and photophobia, which caused him to walk alongside walls for support. After 1 year, he developed difficulty in swallowing, which was gradual in onset and progressive in nature. Initially, dysphagia occurred for liquids and later for solids. This was associated with nasal regurgitation of food and liquids, frequent coughing during meals, nasal tone of voice, and hoarseness. He was treated symptomatically with muscle relaxants, multivitamins, Coenzyme Q‐10, antihistamines, antibiotics, and mometasone nasal spray. He had previously completed a 6‐month course of anti‐tuberculous therapy. No significant history of trauma, congenital malformation, or surgical interventions.

## 3 Differential Diagnosis and Investigations

On general physical examination, no pallor, cyanosis, clubbing, edema, or lymphadenopathy; vital signs were stable with a BP of 110/80 mmHg, pulse 92 beats per minute, respiratory rate 20 breaths per minute, temperature 98.4°F, random blood sugar 120 mg/dL, oxygen saturation 97% on room air, and GCS was 15/15.

On neurological examination there was increased tone on the right side with confirmation of right lower limb clonus and fasciculations, power was 4/5 grade in both right upper and lower limbs, and deep tendon reflexes were brisk (+3) showing on the right side. Cerebellar examination showed an ataxic gait and positive finger‐to‐nose dysmetria, more marked on the right side. Cranial‐nerve examination demonstrated multidirectional nystagmus, optic atrophy with decreased visual acuity, diminished gag reflex, uvular deviation to the left, nasal speech, and decreased movement of the right soft palate. Fundoscopy confirmed optic atrophy with blurred disc margins.

### 3.1 Neuroimaging

MRI of the brain and cervical spine revealed herniation of the cerebellar tonsils extending approximately 10.3 mm below the foramen magnum (Figure 1), consistent with Arnold–Chiari Malformation Type II, along with a syrinx extending through the spinal cord, suggestive of syringomyelia. The syrinx involved the cervical and upper thoracic cord, extending from the C2 to the T1 vertebral level (a longitudinal span of approximately 10 cm), and reached a maximal axial diameter of about 6 mm at the C5–C6 level. The cavity was predominantly central, non‐septated, and showed no surrounding cord edema or pre‐syrinx signal change. No intramedullary mass, hemorrhage, or contrast enhancement was identified.

**FIGURE 1:**
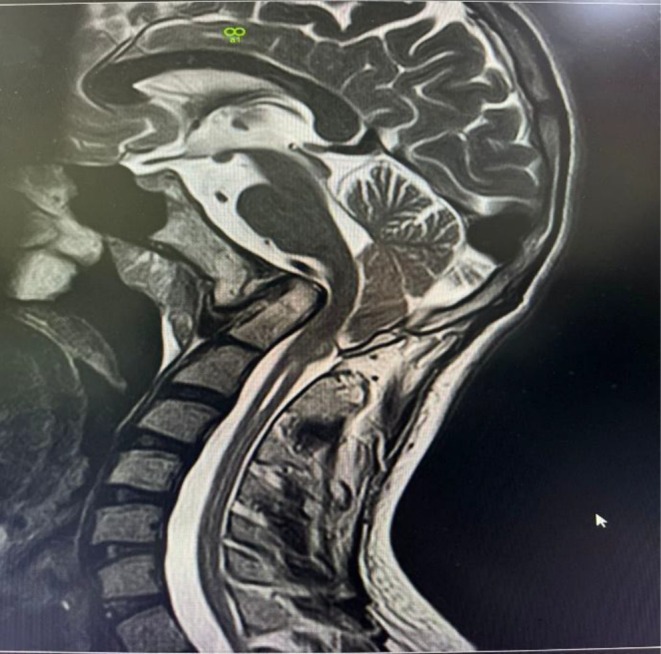
MRI demonstrating Arnold–Chiari Malformation Type II, along with a syrinx extending through the spinal cord.

CT brain, shown in Figure 2 highlights basilar invagination with the odontoid tip lying 1.0 cm above Chamberlain's line and descent of the cerebellar tonsils 1.5 cm below the McRae line, without any encephalocele or myelomeningocele. CT chest revealed a tracheoesophageal fistula, above the carina measuring 0.5 cm in diameter, with a small 2 mm tracheal diverticulum communicating with the esophagus.

**FIGURE 2:**
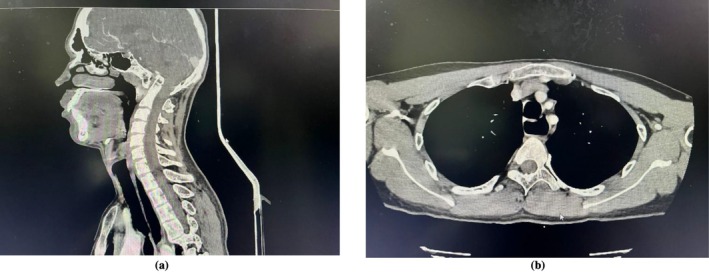
CT scan showing (a) descent of the cerebellar tonsils, (b) Tracheoesophageal fistula above the carina.

### 3.2 Laboratory Tests

Laboratory investigations were largely unremarkable and did not contribute to clinical decision‐making. The complete blood count showed a mild microcytic, hypochromic anemia (hemoglobin 11.4 g/dL; MCV 71.8 fL; MCH 21.0 pg) with a normal red cell count. Serum electrolytes revealed minor derangements only, with a mildly elevated plasma sodium of 148.9 mmol/L and a mildly low plasma potassium of 3.32 mmol/L; arterial blood gases (pCO_2_ 38.1 mmHg, pO_2_ 92.4 mmHg) were normal. Liver function tests were unremarkable apart from a modest elevation of alkaline phosphatase (267 U/L) with a normal gamma‐glutamyl transferase, suggesting a non‐cholestatic, likely bone‐origin pattern. Total bilirubin and prothrombin time (11.4 s) were within the reference range. Full details are provided in Table 1.

**TABLE 1:** Laboratory profile in Arnold–Chiari Malformation Type II with tracheoesophageal fistula: Diagnostic findings and treatment‐monitoring parameters.

Laboratory test	Result	Normal range
Hemoglobin	11.4 g/dL	14.0–17.4 g/dL
MCV	71.8 fL	76.5–96.0 fL
MCH	21.0 pg	27.0–32.0 pg
RBC count	5.43 × 10^12^/L	4.00–5.50 × 10^12^/L
Plasma sodium	148.9 mmol/L	136–145 mmol/L
Plasma potassium	3.32 mmol/L	3.5–5.10 mmol/L
pCO_2_	38.1 mmHg	32.0–48.0 mmHg
pO_2_	92.4 mmHg	83.0–108.0 mmHg
Total bilirubin	0.4 mg/dL	0.3–1.2 mg/dL
Alkaline phosphatase	267 U/L	30–120 U/L
Gamma‐GT	16 U/L	< 55 U/L
Prothrombin time	11.4 s	9.92–12.88 s

## 4 Treatment

Patient was referred to a neurosurgical and thoracic surgeon. He underwent posterior fossa decompression surgery, including suboccipital craniectomy and C1 laminectomy, to relieve compression at the foramen magnum and restore cerebrospinal fluid flow. Synchronously, thoracic surgeons repaired the tracheoesophageal fistula with reinforcement of the esophageal wall. Postoperatively, the patient was managed with nutritional support, a nasogastric tube to prevent aspiration, and speech and swallowing rehabilitation supervised by a speech‐language pathologist. Progressive recovery, with improved swallowing ability, reduced aspiration, and strengthened gait stability through physiotherapy, was observed.

The major clinical events, including diagnostic findings and treatment interventions, are summarized in Table 2.

**TABLE 2:** Timeline of clinical events.

Time point	Clinical event
2 years prior	Gradual onset of right‐sided weakness and numbness
1.5 years prior	Development of imbalance, dizziness, and headaches; vertigo and photophobia also noted
1 year prior	Onset of dysphagia, nasal regurgitation, hoarseness, and coughing during meals
6 months prior	Completed anti‐tuberculous therapy and received symptomatic management
At presentation	Progressive right‐sided weakness, ataxia, nasal tone, and optic atrophy observed
Investigations	MRI and CT scan revealed Arnold–Chiari Malformation Type II and tracheoesophageal fistula
Treatment	Posterior fossa decompression (suboccipital craniectomy + C1 laminectomy) and TEF repair performed

## 5 Conclusion and Results

On 6‐month follow‐up, MRI revealed a decrease in crowding at the foramen magnum and stabilization of the syrinx. The tracheoesophageal fistula was fully obliterated, and the patient showed progress in oral feeding without regurgitation or coughing. There was a significant reduction in vertigo and imbalance. The patient remains under regular follow‐up with neurologists, neurosurgeons, ENT specialists, and pulmonologists to ensure continued care and early detection of any recurrence or neurological deterioration.

ACM II is a neurological disorder most commonly identified in neonates. Its presentation in an adult, as described here, is rarely documented. In our patient, the condition was associated with a tracheoesophageal fistula, syringomyelia, and progressively worsening craniospinal and bulbar symptoms. This case emphasizes the importance of thorough neuroimaging, cross‐specialty collaboration, and a high index of clinical suspicion when classic pediatric hindbrain malformations present in adulthood.

## 6 Discussion

Type II ACM identification in a young adult, as seen in this case, is very rare since it usually presents in neonates and emphasizes the need for heightened clinical awareness in such patients with unusual or progressive craniospinal presentation. This case is further unique due to the association of TEF, a defect typically identified in neonates, but identified during adulthood in our patient. Hence, the simultaneous co‐existence of ACM and TEF highlights the hypothesis of a shared embryological or developmental pathway, connecting hindbrain malformation with foregut anomalies.

The embryological correlation suggests that ACM and TEF originate from the aberrant mesodermal development in the foregut and neural tube development during 3rd and 6th weeks of gestation [13]. Due to the interruption in mesodermal signaling pathways, including mutation in HOX gene expression, dysregulation of Sonic Hedgehog (SHH) pathway, or disruption in neural crest cell migration, leads to posterior cranial fossa malformation causing ACM and defective septation of trachea and esophagus leading to TEF. In addition, during early embryogenesis, disruption in cerebrospinal fluid (CSF) dynamics may raise intracranial pressure, leading to defective foregut positioning and altered neural tube closure leading to ACM [14].

Although ACM Type II typically presents in early life, late presentation in adults can be ascribed to progressive tonsillar descent, secondary CSF flow obstruction, or late diagnosis because of overlapping symptomatology. As in this case, management of TEF is necessary by either surgical repair or multidisciplinary program of neurosurgery, thoracic surgery, and otolaryngology [15]. Diagnostic delay may have also been caused by our patient's history of pulmonary tuberculosis and treatment, which would have masked or obscured early neurological signs. The tracheoesophageal fistula was classified as acquired rather than congenital on the basis of several converging features. The patient had no infantile feeding difficulties, recurrent aspiration pneumonias in childhood, or growth failure that would be expected with an untreated congenital TEF, and there was no prior surgical record. Symptoms of dysphagia, nasal regurgitation, and coughing during meals appeared only 1 year before presentation, against a background of recently treated pulmonary tuberculosis. Pulmonary tuberculosis is a recognized cause of acquired non‐malignant TEF through transmural caseating necrosis, peri‐tracheal lymphadenopathy, or post‐treatment fibrosis [10, 15]. The lesion morphology on imaging—a small, single 0.5 cm communication just above the carina with an adjacent 2 mm tracheal diverticulum—is more consistent with an inflammatory or post‐infectious fistula than with the broader, more proximal defects typical of congenital H‐type TEF. Congenital H‐type TEF, when it does escape neonatal detection and present later in life, has several characteristic features that were absent in our patient: a lifelong history of paroxysmal coughing on swallowing liquids, recurrent unexplained pulmonary infections, bronchiectasis or chronic lung damage from chronic aspiration, and a typical location in the upper third of the trachea, with the tracheal opening lying obliquely cephalad to the esophageal opening, giving the lesion its characteristic “H” configuration [16]. Our patient had a normal childhood feeding history and no chronic respiratory symptoms preceding the recent tuberculous illness, the fistula was located low in the trachea immediately above the carina, and the imaging showed a small horizontal communication rather than the classical oblique tract. Taken together, the temporal sequence, infectious history, and imaging features support an acquired etiology in this patient.

MRI confirmed a 10.3 mm herniation of the tonsils below the foramen magnum and a coincidental syringomyelia, which in up to 70% of ACM cases is caused by disturbance of CSF dynamics [17]. Syringomyelia also accounts for his sensorimotor deficits, fasciculations, and worsening neurological course which mandates close observation, as syrinxes left untreated can lead to irreversible deficit. Classification as Chiari II in this patient was supported not by tonsillar descent alone, but by the combination of caudal displacement of the cerebellar tonsils and vermis below the foramen magnum, downward elongation of the medulla and fourth ventricle, syringomyelia, basilar invagination, and bulbar cranial‐nerve dysfunction (palatal weakness, diminished gag, nasal speech). By contrast, Chiari 1.5 is defined as tonsillar ectopia together with caudal displacement of the brainstem (in particular the obex) but without vermian descent, fourth ventricle elongation, or the additional hindbrain‐stem distortion that characterizes Chiari II; complex Chiari refers principally to Chiari I with associated craniocervical bony anomalies such as basilar invagination or atlanto‐axial instability [3]. In our patient the imaging showed not only tonsillar herniation but also descent of the vermis and elongation of the medulla and fourth ventricle below the foramen magnum, together with bulbar cranial‐nerve compromise—findings that go beyond Chiari I, Chiari 1.5, or purely bony “complex Chiari” phenotypes. We acknowledge that the absence of a myelomeningocele is atypical for Chiari II, and that some authors would prefer the descriptive term “complex Chiari” or “Chiari 1.5” in such cases [3]. We have retained the Chiari II designation because the constellation of hindbrain and brainstem findings extends beyond isolated tonsillar ectopia, but we recognize that a small minority of adult cases without spinal dysraphism may be reclassified as more is learned about late‐presenting hindbrain malformations.

The wide differential diagnosis in this type of presentation involves brainstem glioma, multiple sclerosis, myasthenia gravis, demyelinating neuropathies, and sequelae of CNS tuberculosis. But the presence of structural hindbrain herniation, syrinx formation, cranial‐nerve involvement, and airway‐esophageal communication rendered ACM Type II as the unifying diagnosis. This case underscores the indispensable role of neuroimaging in complicated craniospinal syndromes, especially when extracranial manifestations are present [1].

A particularly instructive aspect of this case is the 21‐year diagnostic delay. Early subtle symptoms such as right‐sided weakness, imbalance, dizziness, and chronic headache had been attributed to the patient's pulmonary tuberculosis and were managed symptomatically with antihistamines, antibiotics, muscle relaxants, and vestibular suppressants without dedicated neuroimaging. The later development of bulbar features—nasal regurgitation, hoarse voice, coughing during meals, and a nasal speech quality—should be regarded as “red flag” symptoms because they cannot be explained by pulmonary tuberculosis alone. Of particular note, as outlined in the timeline of clinical events (Table 2), these bulbar features emerged approximately 1 year after the initial motor symptoms; this interval between the motor and bulbar phases is itself an important clue, indicating a progressive structural lesion rather than a static or purely infective process, and it marks the point at which the diagnosis was most clearly missed. In adult Chiari patients, dysphagia, dysarthria, and lower cranial‐nerve dysfunction are strongly associated with bulbar or upper cervical syringomyelia and frequently improve after posterior fossa decompression [18]. We therefore propose that the new appearance of bulbar symptoms in any adult, including those with active or recently treated tuberculosis, should prompt MRI of the brain and craniocervical junction to exclude structural hindbrain pathology rather than attributing the symptoms to the pre‐existing infectious diagnosis.

## Author Contributions

**Zahabia Adnan:** conceptualization, formal analysis, supervision, visualization, writing – original draft, writing – review and editing. **Yashfa Rajput:** conceptualization, project administration, writing – original draft, writing – review and editing. **Chanchan:** methodology, writing – original draft. **Marium Fawad:** investigation, writing – original draft, writing – review and editing. **Abedin Samadi:** writing – original draft, writing – review and editing. **Ahmed Asad Raza:** project administration, supervision, writing – original draft, writing – review and editing. **Muhib Ullah Azhar:** project administration, supervision, validation, writing – review and editing.

## Funding

The authors have nothing to report.

## Ethics Statement

The authors have nothing to report.

## Consent

The patient gave a written informed consent to participate in the study and use their anonymized information. The patient is an adult (21 years of age) and provided his own written informed consent for the publication of this case report and any accompanying images.

## Conflicts of Interest

The authors declare no conflicts of interest.

## Data Availability

The data supporting the findings of this study are available from the corresponding author upon reasonable request. Due to the nature of the study, patient confidentiality and ethical considerations prevent the public sharing of sensitive clinical data. All other data related to the study, including de‐identified information, can be made available upon request, in accordance with the journal's data‐sharing policies.
